# *Geobacillus stearothermophilus* 6-phosphogluconate dehydrogenase complexed with 6-phosphogluconate

**DOI:** 10.1107/S1744309109012767

**Published:** 2009-04-24

**Authors:** Scott Cameron, Viviane P. Martini, Jorge Iulek, William N. Hunter

**Affiliations:** a Division of Biological Chemistry and Drug Discovery, College of Life Sciences, University of Dundee, Dundee DD1 5EH, Scotland; b Universidade Estadual de Ponta Grossa, Departamento de Química, Avenida Carlos Cavalcanti, 4748 Uvaranas, 84030-000 Ponta Grossa, Paraná, Brazil

**Keywords:** pentose phosphate pathway, 6-phosphogluconate dehydrogenase, *Geobacillus stearothermophilus*

## Abstract

Two crystal structures of recombinant *Geobacillus stearothermophilus* 6-phosphogluconate dehydrogenase (*Gs*6PDH) in complex with the substrate 6-­phosphogluconate have been determined at medium resolution. *Gs*6PDH shares significant sequence identity and structural similarity with the enzymes from *Lactococcus lactis*, sheep liver and the protozoan parasite *Trypanosoma brucei*, for which a range of structures have previously been reported. Comparisons indicate that amino-acid sequence conservation is more pronounced in the two domains that contribute to the architecture of the active site, namely the N-terminal and C-terminal domains, compared with the central domain, which is primarily involved in the subunit–subunit associations required to form a stable dimer. The active-site residues are highly conserved, as are the interactions with the 6-phosphogluconate. There is interest in 6PDH as a potential drug target for the protozoan parasite *T. brucei*, the pathogen responsible for African sleeping sickness. The recombinant *T. brucei* enzyme has proven to be recalcitrant to enzyme–ligand studies and a surrogate protein might offer new opportunities to investigate and characterize 6PDH inhibitors. The high degree of structural similarity, efficient level of expression and straightforward crystallization conditions mean that *Gs*6PDH may prove to be an appropriate model system for structure-based inhibitor design targeting the enzyme from *Trypanosoma* species.

## Abstract 2

The structure of 6-phosphogluconate dehydrogenase from a moderate thermophile, *G. stearothermophilus*, is presented and compared with those of orthologous enzymes.

## 1. Introduction

The enzyme 6-phosphogluconate dehydrogenase (6PDH; EC 1.1.1.44) catalyzes the conversion of 6-phosphogluconate (6PG) to ribulose 5-phosphate (R5P), a reaction that occurs in the pentose phosphate pathway that generates NADPH and pentose sugars (Barrett, 1997 ▶). This is an essential activity in eukaryotic cells (Lobo & Maitra, 1982 ▶; Hughes & Lucchesi, 1977 ▶) and the deleterious effects generated by compromising 6PDH activity have been ascribed to the disruption of glycolysis by 6PG inhibition of phosphoglucose isomerase (Marchand *et al.*, 1989 ▶). The essential nature of 6PDH suggests that it is a potential drug target in *Trypanosoma brucei*, a protozoan parasite that is responsible for African sleeping sickness (Barrett, 1997 ▶). Further support for this conclusion comes from the observation that selective inhibition of the parasite over mammalian enzymes appears to be possible (Hanau *et al.*, 2004 ▶). The structures of 6PDH from sheep (*Oa*6PDH; Adams *et al.*, 1994 ▶), *T. brucei* (*Tb*6PDH; Phillips *et al.*, 1998 ▶), *Lactococcus lactis* (*Ll*6PDH; Sundaramoorthy *et al.*, 2007 ▶) and *Saccharomyces cerevisiae* (He *et al.*, 2007 ▶) have been reported.

Our studies with 6PDH from a variety of species, in particular the *T. brucei* enzyme, have proven to be problematic, with limitations in protein stability and solubility compounding difficulties in structural studies. The enzyme from *L. lactis* enabled us to generate some data on protein–ligand complexes (Tetaud *et al.*, 1998 ▶; Sundaramoorthy *et al.*, 2007 ▶). However, this enzyme does not produce good-quality crystals in a reproducible fashion and the presence of one long unit-cell length (>240 Å) renders it difficult to routinely measure diffraction data to high resolution. An alternative source of suitable crystals was sought to provide a surrogate model to assist structure-based ligand studies that guide the development of novel inhibitors (Hunter, 2009 ▶). The crystallization of 6PDH from the moderate thermophile *Geobacillus stearothermophilus* has been reported (*Gs*6PDH; Pearse & Harris, 1973 ▶) and we sought to establish whether this particular enzyme would provide a useful source of crystals. We now report the preparation of an efficient recombinant protein-production system, crystallization, structure determination and comparisons with previously determined structures.

## 2. Methods and materials

### 2.1. Cloning, expression and purification

The gene encoding 6PDH was identified using a *BLAST* search (Altschul *et al.*, 1990 ▶), with sequence information based on 6PDH from *L. lactis* (accession No. NP_266778), against the unfinished *G. stearothermophilus* genome (http://www.genome.ou.edu/bstearo.html). Genomic DNA from *G. stearothermophilus* (American Type Culture Collection 12980) was used as template for PCR with the following primers designed to amplify the 6PDH open reading frame and incorporating *Nde*I and *Xho*I restriction sites (bold), respectively: 5′-­**CAT**-**ATG**-GCT-AAA-CAG-CAA-ATC-GGC-G-3′ and 5′-**CTC**-**GAG**-TTA-TTT-CAA-CCA-TTC-CGT-ATG-3′. The PCR product was ligated into pCR-BluntII-TOPO vector using the Zero Blunt TOPO PCR cloning kit (Invitrogen). Once identified, the gene was excised with *Nde*I/*Xho*I and then ligated into a modified pET15b vector (Novagen) in which the sequence that encodes a thrombin protease cleavage site had been replaced with a tobacco etch virus (TEV) protease recognition site. This plasmid therefore encodes a product with an N-terminal hexahistidine tag, which is removed by TEV protease. The plasmid was amplified in XL-1 Blue *Escherichia coli* and the integrity of the PCR product was verified by sequencing and then used to transform *E. coli* strain BL21 (DE3) pLysS for protein production.

Bacteria were cultured with shaking at 200 rev min^−1^ in autoinduction media (Studier, 2005 ▶) with 50 mg l^−1^ carbenicillin for approximately 2 h at 310 K followed by 22 h at 295 K. Cells were harvested by centrifugation (3500*g*, 20 min, 277 K) and resuspended in 50 m*M* Tris–HCl pH 7.4, 250 m*M* NaCl. DNAse I (100 µg) and an EDTA-free protease-inhibitor cocktail tablet (Roche) were added to the mixture before the cells were lysed in a TS-75 cell disruptor (Constant Systems) and the soluble fraction was isolated by centrifugation (50 000*g*, 30 min, 277 K). The cell extract was filtered and applied onto a HisTrap HP 5 ml column (GE Healthcare). The His-tagged protein was eluted with a 0–1 *M* imidazole gradient in the same buffer. Histidine-tagged TEV protease (1 mg in the same buffer per 20 mg 6PDH) was added to the product and the mixture was dialysed against the same buffer as used for cell lysis. Passage over a His-Trap column removed the TEV protease, the cleaved His-tag peptide and any 6PDH which retained the tag and the processed 6PDH was collected. A final purification step involved gel filtration (Superdex 200, 26/60 column, GE Healthcare) before buffer exchange into 50 m*M* Tris–HCl pH 7.4, 20 m*M* NaCl and concentration to 12 mg ml^−1^ for crystallization trials. The high level of protein purity was confirmed by SDS–PAGE and matrix-assisted laser desorption ionization–time-of-flight mass spectrometry. The yield of purified protein was approximately 20 mg per litre of bacterial culture.

### 2.2. Crystallization, data collection and processing

Crystals grew over 2 d in hanging drops consisting of 1 µl protein solution and 1 µl reservoir solution (0.2 *M* lithium sulfate, 2.2 *M* ammonium sulfate or 0.2 *M* ammonium acetate, 2.2 *M* ammonium sulfate) equilibrated against 500 µl reservoir solution at 293 K. Crystals were also obtained in the presence of 2 m*M* 6PG and 2 m*M* NADP^+^ or 2 m*M* R5P and 2 m*M* NADP^+^. Single crystals (blocks with approximate dimensions 0.2 × 0.2 × 0.2 mm) were placed in a nylon loop (Hampton Research), mounted on a goniostat, cooled to 100 K with gaseous nitrogen and then characterized using a Micromax-007 rotating-anode generator and an R-AXIS IV^++^ dual image-plate detector (Rigaku). Suitable crystals were stored in liquid nitrogen and X-ray diffraction data were then measured with a wavelength of 0.979 Å and a Q315r charge-coupled device detector (Area Detector Systems Corporation) on beamline ID14-4 of the European Synchrotron Radiation Facility. Although all crystals were isomorphous, those grown in the presence of lithium sulfate diffracted to higher resolution than those obtained with sodium acetate and so they were used for the analyses. Two data sets were obtained: data set I from a crystal (hereafter called crystal I) grown in the presence of 6PG which diffracted to 2.3 Å resolution and data set II acquired from two crystals grown in the presence of R5P which diffracted to 2.2 Å resolution. The subsequent analysis of data set II revealed only the presence of 6PG in the corresponding crystals.

Diffraction data from crystal I were processed using *MOSFLM*/*SCALA* (Leslie, 2006 ▶; Evans, 2006 ▶), whilst data set II was generated using *XDS*/*XSCALE* (Kabsch, 2001 ▶). Relevant results are shown in Table 1 ▶.

### 2.3. Molecular replacement, model building and refinement

The data sets were processed and the structures solved and refined independently, but the same *R*_free_ set was maintained. Both a polyalanine model and mixed model, with conserved residues left as they are, based on an *L. lactis* 6PDH subunit (PDB code 1iyo) were constructed using *CHAINSAW* from the *CCP*4 suite of programs (Collaborative Computational Project, Number 4, 1994 ▶). These two proteins share 66% sequence identity. These models were used for molecular-replacement calculations in *Phaser* (McCoy *et al.*, 2005 ▶) to position the two subunits that constitute the asymmetric unit. Refinement was carried out using *REFMAC*5 (Murshudov *et al.*, 1997 ▶) interspersed with electron-density and difference density map inspection, model manipulation and the incorporation of solvent and ligands using *Coot* (Emsley & Cowtan, 2004 ▶). The asymmetric unit consists of two monomers and they were treated independently during refinement. Crystallographic statistics are given in Table 1 ▶. Figures were prepared with *PyMOL* (http://www.pymol.org) and *ALINE* (Bond & Schüttelkopf, 2009 ▶).

## 3. Results and discussion

### 3.1. Structure determination

Two isomorphous crystal structures were determined (Table 1 ▶). Data set I was obtained from a sample grown in the presence of 6PG and NADP^+^ and data set II was obtained from crystals grown in the presence of R5P and NADP^+^. In neither structure was there electron density that could be attributed to NADP^+^. In structure I, 6PG is present in both active sites of the dimer that constitutes the asymmetric unit. However, structure II contained only 6PG, not R5P, and then only in one active site with a reduced occupancy of 0.7. This value was assigned following several rounds of refinement testing different levels of occupancy to elucidate that which gave clean difference density maps and thermal parameters consistent with the amino-acid side chains in the vicinity of the ligand. We have no definitive explanation of why we only see partially ordered 6PG in one active site of structure II, although we note a difference in the time that elapsed between crystal growth and cryo-freezing. Crystal I was frozen and stored in liquid nitrogen within one week of setting up the crystallization conditions. The crystals used to generate data set II were frozen after a period of two months.

A pronounced similarity of the two subunits in the asymmetric units of both crystals is observed despite not employing noncrystallographic symmetry restraints during refinement. The overlay of 467 C^α^ positions of structure I subunit *A* on subunit *B* results in an r.m.s.d. of 0.53 Å; for structure II an r.m.s.d. of 0.70 Å is observed from the overlay of 470 C^α^ atoms. The models derived from the independent analyses are also highly conserved. The overlay of the structure I and structure II dimers, 935 C^α^ atoms, gives an r.m.s.d. of 0.29 Å. In both structures Lys3, Gly137, Asp175 and Thr453 of both subunits are in disallowed regions of the Ramachandran plot.

To all intents, the protein structures are identical. The only significant difference is that structure II only has 6PG in a single active site and at a reduced occupancy. We therefore detail structure I.

### 3.2. Overall structure

**Figure 1:**
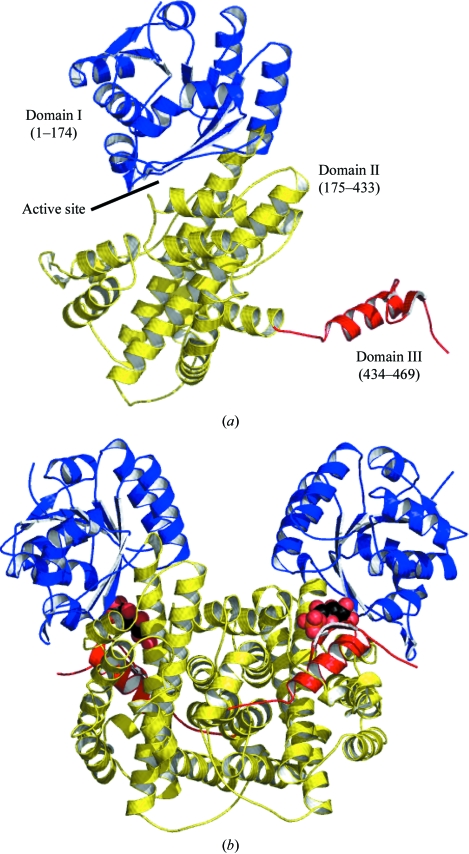
Secondary and domain structure of *Gs*6PDH. (*a*) A ribbon diagram of one subunit. The N-terminal domain is blue, the central domain is yellow and the C-terminal domain is red. (*b*) Ribbon diagram of the dimer, coloured as in (*a*). The ligand 6PG is shown as van der Waals spheres coloured black for C, red for O and orange for P.

*Gs*6PDH shows the same overall fold and three-domain architecture as the other 6PDH structures known (Fig. 1 ▶; Adams *et al.*, 1994 ▶; Sundaramoorthy *et al.*, 2007 ▶). The N-terminal domain, which is formed from the residues up to 174, harbours the site in which NADP^+^ binds. This domain adopts the Rossmann nucleotide-binding domain fold. The central domain is a largely helical domain (ten α-­helices, residues 175–433) that is primarily involved in dimer formation. The C-­terminal domain or ‘tail domain’ consists of a single α-helix followed by two β-strands and extrudes from the central domain deep into the partner subunit. The active site of 6PDH is formed largely by residues contributed from the N-terminal domain but with one side of the cleft lined by residues from the tail domain of the partner subunit; this will be discussed later.

### 3.3. Structural and sequence comparisons

**Figure 2:**
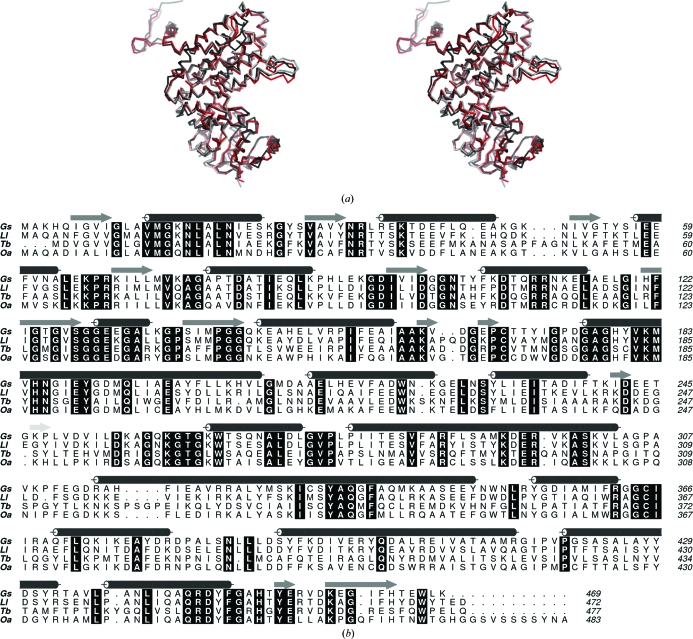
Comparison of the primary and secondary structures of *Gs*6PDH, *Ll*6PDH, *Tb*6PDH and *Oa*6PDH. (*a*) Stereoview C^α^ trace of *Gs*6PDH (grey) superimposed on *Ll*6PDH (black) and *Tb*6PDH (red). (*b*) Sequence alignment of four 6PDH enzymes, with the assigned secondary structure of *Gs*6PDH (cylinders. α-helices; arrows, β-strands). Strictly conserved residues are shown in black boxes.

The *G. stearothermophilus* 6PDH models display high similarity to the *L. lactis*, *O. aries* and *T. brucei* 6PDH structures (Fig. 2 ▶ *a*). The least-squares superpositions of C^α^ positions of subunit *A* of structure I with a subunit of each of these orthologues results in r.m.s.d. values of 1.51 Å (461 residues), 0.90 Å (464 residues) and 1.60 Å (454 residues), respectively.

*Gs*6PDH shares 66% sequence identity with *Ll*6PDH and 57% and 36% identity with *Oa*6PDH and *Tb*6PDH, respectively. A sequence alignment of these four enzymes is presented in Fig. 2 ▶(*b*). About 26% of the residues are strictly conserved across all four sequences; however, the level of conservation varies according to the domain. In the N-terminal, central and tail domains there are 32, 21 and 36% of residues, respectively, that are strictly conserved. If this comparison is restricted to the two bacterial enzymes the values are 66, 63 and 75%, respectively. Comparison of the *Gs*6PDH and *Tb*6PDH sequences reveals values of 49, 26 and 44% for the three domains. The N-terminal and tail domains contribute to the creation of the active site and display a higher level of sequence conservation compared with the central domain, which contributes to dimer formation.

Cofactor binding to 6PDH occurs in a groove on the periphery of the N-terminal domain, with the adenine ribose placed at the β1–α1 turn that carries a nucleotide-binding motif. In *Gs*6PDH the sequence is Gly10-Leu11-Ala12-Val13-Met14-Gly15 and this matches closely with that observed in most organisms: a Gly-*X*-Ala-*X*-*X*-Gly motif. In *Tb*6PDH the motif is altered to Gly-*X*-Gly-*X*-*X*-Gly (Phillips *et al.*, 1998 ▶; Fig. 2 ▶ *b*). At present it is unclear whether this glycine–alanine difference will influence the catalytic properties of the respective enzymes or lead to structural differences when the cofactor is bound. Further structures of both *Tb*6PDH and *Gs*6PDH in complex with the cofactor would be required in order to address this point.

### 3.4. Ligand binding

**Figure 3:**
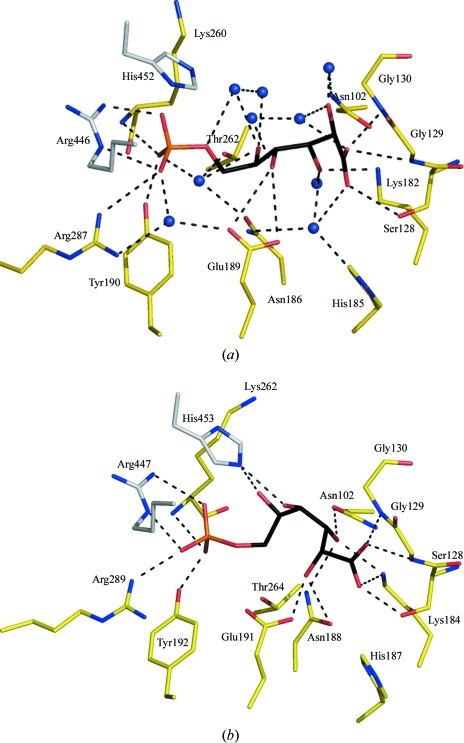
The 6PG-binding site of *Gs*6PDH and comparison with *Ll*6PDH. (*a*) The *Gs*6PDH active site. Atoms are coloured according to type: N, blue; O, red; P, orange; the C atoms of residues associated with subunit *A* are shown in grey, those associated with subunit *B* are shown in yellow and those in the ligand are shown in black. Water molecules are depicted as blue spheres and dashed lines indicate potential hydrogen-bonding interactions. (*b*) The *Ll*6PDH active site in a similar orientation and representation. For the purpose of clarity, water molecules and associated hydrogen-bond interactions have been omitted from this figure.

The interactions formed by 6PG with 6PDH have previously been detailed for the *L. lactis* enzyme and compared with other structures (Sundaramoorthy *et al.*, 2007 ▶). The 6PG substrate-binding site of *Gs*6PDH and the interactions formed with this ligand (Fig. 3 ▶) are highly conserved in other 6PDH structures. The phosphate of 6PG is positioned by accepting hydrogen bonds from Tyr190, Lys260, Arg287 of one subunit and Arg446 of the partner subunit. Ordered water-mediated hydrogen bonds also link the phosphate group to Asn186, Glu189 and Thr262. Additional hydrogen bonds, including several water-mediated interactions, involving His552 from one subunit and Asn102, Ser128, Gly129, Gly130 and Asn186 from the partner hold the rest of the ligand in place. These residues include two that have been shown to be critical for the enzyme mechanism, Lys182 (Zhang *et al.*, 1999 ▶) and Glu189 (Karsten *et al.*, 1998 ▶), and others (for example, Ser128, Asn187 and His186) that are responsible for ligand binding and catalysis (Li, Dworkowski *et al.*, 2006 ▶; Li, Zhang *et al.*, 2006 ▶). All of these 13 residues are strictly conserved in the 6PDH sequences from diverse species ranging from a higher eukaryote to a protozoan eukaryote and two different Gram-positive bacteria. Such a high level of sequence and structural conservation suggests that *Gs*6PDH could serve as an appropriate surrogate for structure-based ligand design targeting the pathogenic protozoan *T. brucei*.

## Supplementary Material

PDB reference: 6-phosphogluconate dehydrogenase, 2w8z, r2w8zsf

PDB reference: 2w90, r2w90sf

## Figures and Tables

**Table 1:** Crystallographic statistics Values in parentheses are for the highest resolution shell bin with a width of 0.06 Å.

	Data set I	Data set II
Space group	*P*2_1_2_1_2_1_	*P*2_1_2_1_2_1_
Unit-cell parameters (Å)	*a* = 67.04, *b* = 119.97, *c* = 142.83	*a* = 66.95, *b* = 119.50, *c* = 142.24
Resolution range (Å)	71.43–2.30	71.07–2.20
No. of unique reflections	52157	57708
Completeness of data (%)	99.9 (99.9)	99.3 (99.6)
〈*I*/σ(*I*)〉	14.1 (3.2)	10.4 (3.6)
*R* factor (%)	18.2 (23.2)	17.1 (26.3)
*R*_free_ (%)	23.4 (30.8)	23.4 (36.3)
R.m.s.d from ideal values		
Bond lengths (Å)	0.012	0.012
Angles (°)	1.389	1.276
Average *B* values (Å^2^)		
Protein	26.0	40.8
Main chain	25.5	39.5
Waters	26.6	42.1
Side chain	26.6	47.1
6PG [occupancy]	75.7 [1.0]	53.7 [0.7]
Wilson *B* (Å^2^)	28.8	31.4
Ramachandran plot analysis†		
Most favoured regions	93.1 [757]	91.8 [752]
Additional allowed regions	6.2 [50]	7.3 [60]
Generously allowed	0.1 [1]	0.4 [3]
Disallowed regions	0.6 [5]	0.5 [4]

† The numbers in square brackets in the Ramachandran analysis are the number of residues in each category.
